# High-throughput identification of immunoreactive peptides and corresponding proteins from *Anaplasma platys* and *Ehrlichia canis* using peptide microarray chips

**DOI:** 10.3389/fcimb.2025.1671309

**Published:** 2026-01-07

**Authors:** Alejandro Llanes, Swetha Madesh, Kalvis Brangulis, Sreekumari Rajeev

**Affiliations:** 1 División de Salud Humana y Enfermedades, Instituto de Investigaciones Científicas y Servicios de Alta Tecnología (INDICASAT-AIP), Panama City, Panama; 2 Sistema Nacional de Investigación (SNI), Panama City, Panama; 3 Department of Biomedical and Diagnostic Sciences, College of Veterinary Medicine, University of Tennessee, Knoxville, TN, United States; 4 Latvian Biomedical Research and Study Centre, Riga, Latvia

**Keywords:** *Anaplasma platys*, immunoreactive peptides, B-cell epitopes prediction, peptide microarrays, high-throughput identification

## Abstract

**Introduction:**

*Anaplasma platys* and *Ehrlichia canis* are rickettsial pathogens infecting dogs, with a worldwide distribution. Both species are obligate intracellular pathogens and colonize bone marrow-derived cells, with coinfections frequently reported in dogs. Although *E. canis* immunodominant proteins have been thoroughly characterized, very few high-throughput studies have been conducted to identify immunogenic proteins from *Anaplasma* spp. In this study, we used a methodology based on peptide microarray chips to identify immunoreactive peptides, either shared or species-specific, in the complete theoretical proteomes of both pathogens.

**Methods:**

B-cell epitopes were predicted in the corresponding proteins from both species and ranked for synthesis on the peptide microarrays. These microarrays were screened with serum samples from antibody-positive dogs, as well as negative control sera from unexposed dogs. Additionally, we assessed the feasibility of integrating evidence gathered at the level of individual peptides to identify potentially immunogenic proteins contributing to the patterns of immunoreactivity observed on microarrays.

**Results:**

Screening of peptide microarrays resulted in complex antibody reactivity patterns against thousands of peptides. After discarding peptides with cross-reactivity to negative control sera, we identified over 1,200 immunoreactive peptides, including ~80 peptides shared between the two species with almost identical sequences. Despite screening linear peptides, we were able to identify proteins previously reported as immunodominant in *E. canis*, some of which contain predominantly conformational epitopes.

**Discussion:**

Our results suggest that a high-throughput strategy based on peptide microarrays is an effective approach for the rapid identification of immunoreactive peptides and the underlying immunogenic proteins. This study provides a foundation for developing novel diagnostic tools and vaccine candidates against *A. platys* and *E. canis*, including potential combined or multivalent formulations targeting both pathogens.

## 1 Introduction

*Ehrlichia* and *Anaplasma* species are tick-borne rickettsial bacteria infecting humans and animals. These obligate intracellular bacteria colonize the cytoplasm of bone marrow-derived cells, including granulocytes, erythrocytes, monocytes, and platelets (Dumler et al., 2001). *Anaplasma platys*, formerly known as *Ehrlichia platys* (Dumler et al., 2001), is unique among known rickettsial pathogens in the fact that it infects platelets and causes infectious canine cyclic thrombocytopenia in dogs (Gaunt et al., 2010). While often mild and subclinical, *A. platys* infections can cause severe, life-threatening disease. *Ehrlichia canis* causes acute, subclinical, and chronic forms of canine monocytic ehrlichiosis (Harrus, 2015). Both pathogens are transmitted by the brown dog tick *Rhipicephalus sanguineus*, and coinfections are common in dogs (Harrus et al., 1997). Belonging to the same *Anaplasmataceae* family (Dumler et al., 2001), both bacterial genera exhibit similarities in their genomic and pathogenic features. Although *E. canis* has been cultured in canine macrophage and tick cell lines (Ferrolho et al., 2016), *A. platys* remains unculturable *in vitro* to date. Both pathogens replicate within parasitophorous vacuoles in host cells, forming structures called morulae. Morulae are readily visible in *A. platys*-infected platelets of acutely infected dogs, but in *E. canis*-infected dogs, they are rarely found in monocytes (Gaunt et al., 2010).

Currently, diagnosis relies on commercially available point-of-care immunochromatography-based antibody tests and indirect immunofluorescence antibody assays (IFA) (Stillman et al., 2014). A few PCR assays are also available for both agents to detect DNA in the blood and tissues of dogs (Sirigireddy and Ganta, 2005; Baneth et al., 2009; Peleg et al., 2010; da Silva et al., 2016; Lara et al., 2020). The increasing impact of tick-borne diseases necessitates effective prevention and control strategies. However, no vaccines currently exist for *E. canis* or *A. platys*. Tick control and antibiotics are the mainstays of prevention and treatment, but the emergence of antibiotic and acaricide resistance is a significant concern (Obaid et al., 2022). The obligate intracellular nature of these pathogens, coupled with the difficulty of *in vitro* culture and establishment of animal models, has hampered vaccine development. Due to this complex scenario, traditional vaccine development strategies may not be optimal and effective for these pathogens. The availability of curated genome sequences and immunoinformatic software tools facilitates the application of the reverse vaccinology paradigm, which is especially useful for pathogens that cannot be grown *in vitro* (Goodswen et al., 2023). This approach uses whole-genome sequences for the identification of potential unique or shared prospective targets to explore as vaccine candidates, some of which may also be valuable for pathogen detection.

Reverse vaccinology strategies begin with the genome-wide prediction of immunogenic proteins containing B-cell or T-cell epitopes, which are then experimentally validated and assayed for protective immune responses. Immunogenic proteins from *E. canis* and other *Ehrlichia* species have been well characterized, and their specific B-cell epitopes have also been identified and experimentally validated in many cases. The first immunoreactive proteins characterized for these species were tandem repeat proteins (TRPs) (McBride et al., 2007; Luo et al., 2009), ankyrin-repeat-containing proteins (Nethery et al., 2007; Luo et al., 2010), and outer membrane proteins (OMPs) (Ohashi et al., 1998a, Ohashi et al., 1998b), all of which have predominantly linear B-cell epitopes. All of these proteins have been shown to elicit strong antibody responses in dogs and humans, and some of them seem to induce protective immune responses. In a series of very comprehensive studies, the genomes of *E. chaffeensis* and *E. canis* were further examined to identify additional immunodominant proteins using a high-throughput approach focused on conformational, rather than linear B-cell epitopes (Luo et al., 2020; Luo et al., 2021; Luo et al., 2023). These studies collectively identified over 50 new immunodominant proteins from both species, most of them with predominantly conformational epitopes, although proteins from *E. canis* were found to have a higher preference for linear epitopes.

Unlike those from *Ehrlichia* spp., less is known about immunogenic proteins from *Anaplasma* species. Most of the studies addressing this topic have focused mainly on *A. phagocytophilum*, a pathogenic species affecting dogs, cats, and horses, and occasionally causing zoonotic disease in humans. Proteins encoded by genes from the very polymorphic *p44/msp2* family have been identified as the major immunodominant surface antigens in all *Anaplasma* species, including *A. platys* (Lin et al., 2004; Lai et al., 2011). A member of this family from *A. phagocytophilum*, MSP4, has been found to interact with host cells during infection (Contreras et al., 2017), and chimeric antigens derived from this protein have also been tested as potential vaccine candidates (de la Fuente et al., 2022; Moraga-Fernández et al., 2024). However, very few studies have implemented high-throughput strategies to identify additional immunogenic proteins, and these have also focused mainly on *A. phagocytophilum* (Ge and Rikihisa, 2007; He et al., 2018). Our group recently sequenced and annotated the *A. platys* genome (Llanes and Rajeev, 2020). Preliminary analysis revealed numerous shared orthologous genes between *A. platys* and *E. canis*, suggesting the possible presence of common or shared epitopes and the possibility of developing a common multivalent vaccine. Here, we used a high-throughput strategy based on peptide microarray chips to identify peptides acting as B-cell epitopes from *A. platys* and *E. canis* proteins, either species-specific or shared between the two pathogens. This information is further used to characterize the underlying immunogenic proteins providing these immunoreactive peptides.

## 2 Materials and methods

### 2.1 In silico B-cell epitope prediction

The genomes of *A. platys* strain S3 (Llanes and Rajeev, 2020) and *E. canis* strain Jake (Mavromatis et al., 2006) were downloaded from the GenBank database under BioProjects PRJNA578763 and PRJNA10694, respectively. The amino acid sequences of all the protein-coding genes encoded by the *A. platys* (*n* = 850) and *E. canis* (*n* = 925) genomes were extracted and subsequently screened with Bepipred v.2.0 (Jespersen et al., 2017). For a single protein sequence, Bepipred assigns each amino acid residue a score that represents the probability of the residue being a part of a linear B-cell epitope. We then selected all the contiguous segments of 9-17 amino acid residues from the predictions for each species, for which the average score was above the 0.5 threshold recommended by Bepipred’s authors. The 9-17 length range was used in this study to ensure adequate synthesis and immobilization on peptide microarray chips in subsequent steps. The selected peptides from both species were then merged together and clustered on the basis of their shared sequence similarity using BLAST v.2.11.0 (Altschul et al., 1990) with a percent identity threshold of 90%. In this study, peptides were labelled after the identifier of the corresponding protein, according to the order in which they were predicted by Bepipred. For example, peptide Ecaj_0017_0019 was the 19^th^ peptide predicted by Bepipred in the sequence of protein Ecaj_0017, and it does not necessarily start at position 19 in the sequence.

### 2.2 Peptide microarray preparation and screening

To validate the in silico B-cell epitope predictions, we evaluated the immunoreactivity of target peptides using a peptide microarray chip treated with canine sera positive for *E. canis* and *A. platys* antibodies. The canine serum samples were collected from a small Caribbean island (Saint Kitts) endemic for *Ehrlichia canis*, *Anaplasma platys*, and their vector *Rhipicephalus sanguineus* in dogs. The samples were confirmed for the presence of antibodies using the SNAP 4Dx test (IDEXX, Westbrook, MN, US). This test is specifically designed to detect exposure to *Ehrlichia* spp. (*E. canis*, *E. ewingii*, and *E. chaffeensis*) and *Anaplasma* spp. (*A. phagocytophilum* and *A. platys*), along with other canine pathogens. The assay uses genus-specific antigens, and there is no cross-reactivity between *Ehrlichia* and *Anaplasma* in this platform. On the island where samples were collected, *Ehrlichia* spp. other than *E. canis* and *Anaplasma* spp. other than *A. platys*, as well as their respective vectors, have not been documented. Therefore, positive animals can reasonably be confirmed as exposed to *E. canis* and *A. platys*. Three serum pools (S1, S2, S3) were created by combining three positive serum samples with varying reactivity in each group. The IgG fraction of each pooled serum sample was affinity-purified using NAbTM Protein A/G columns (Thermo Scientific, Rockford, IL). A negative serum sample collected from unexposed beagle dogs housed for unrelated experiments was used as a negative control. The custom peptide microarray chip was designed to include the 5,000 top-scoring shared and species-specific peptides from previous predictions, printed in duplicate in each chip. The peptide microarray preparation and screening were outsourced to a commercial company (PEPperPRINT, Heidelberg, Germany). Three identical copies of the custom microarray chip were prepared to perform the experiments with each of the serum pools. Each peptide microarray, framed with Influenza virus hemagglutinin (HA) and poliovirus peptides as controls, was initially stained with the secondary antibody to assess background-level interactions that could interfere with the assays. The microarrays were then incubated with the purified IgG fractions at a concentration of 1.5 µg/ml, followed by staining with a fluorescent canine anti-IgG antibody. Microarray read-out was performed with an InnoScan 710-IR Microarray Scanner (Innopsys, Carbonne, France) at scanning gains of 50/10 (red/green). The additional HA and poliovirus control peptides framing the peptide microarrays were stained with murine control antibodies as internal quality control to confirm assay performance and peptide microarray integrity. Microarray image analysis was conducted with PepSlide Analyzer v.1.4 (SICASYS Software, Heidelberg, Germany), using an algorithm that reads raw, foreground, and background fluorescence intensities of each spot, and calculates average median foreground intensities and spot-to-spot deviations for spot duplicates. A maximum spot-to-spot deviation of 40% was tolerated, above which the spot intensity of the corresponding peptide was set to zero.

### 2.3 Ortholog clustering and prediction of protein structure and function

Orthologs among *A. platys* and *E. canis* proteins were clustered using OrthoMCL v.2.0.9 (Li et al., 2003). Ortholog groups with members belonging to the same gene family were further clustered together by using functional information, when available. Large-scale sequence similarity among members of these clustered ortholog groups was verified with MAFFT v.7.49 (Katoh and Standley, 2013) before conducting the additional clustering step. The 3D structure of proteins of interest was modeled using AlphaFold v.3.0 (Abramson et al., 2024). For further structural analysis, only the segments of models containing proper secondary structure elements were selected, excluding large unstructured loops and considering a threshold of predicted local distance difference test (plDDT) score above 50, whenever possible. Conserved functional domains in proteins of interest were identified with Interproscan v.5.74 (Jones et al., 2014), synchronized with InterPro v.105.0. Possible transmembrane topology was inferred by using the TMHMM and Phobius modules, also implemented as part of InterProScan. Additional predictions of signal peptide and membrane topology were performed with SignalP-6.0 (Teufel et al., 2022) and Topcons (Tsirigos et al., 2015). Structural similarity search against the AlphaFold database was performed using the DALI web server (Holm, 2022).

### 2.4 Overlapping peptide analysis

The reactivity of all possible 15-mer overlapping peptides from 10 selected immunogenic proteins (ANPL_02100, Ecaj_0017, Ecaj_0113, Ecaj_0174, Ecaj_0213, Ecaj_0293, Ecaj_0636, Ecaj_0770, Ecaj_0840, and Ecaj_0917) was measured in a custom peptide microarray chip, prepared using the same protocol described in Section 2.2. Briefly, the sequence of each protein was elongated with neutral GSGSGSG linkers at the N- and C-terminus to avoid truncated peptides, and the elongated protein sequences were converted into 15-mer peptides with peptide-peptide overlaps of 14 amino acids. These peptides were printed in duplicate on a chip and were framed by additional HA control peptides. The microarray was incubated with serum sample S1 at a concentration of 1.5 µg/ml, followed by staining with a fluorescent canine anti-IgG antibody. Microarray read-out and image analysis were performed as described in Section 2.2. Median foreground intensities and spot-to-spot deviations were calculated for spot duplicates. A maximum spot-to-spot deviation of 40% was tolerated, above which the spot intensity of the corresponding peptide was set to zero.

## 3 Results

### 3.1 *In silico* prediction of potential B-cell epitopes

The first goal of this study was to identify shared or potentially species-specific B-cell epitopes in *A. platys* and *E. canis* proteins. The sequences of 850 and 925 proteins, respectively comprising the complete theoretical proteomes of *A. platys* and *E. canis*, were screened with Bepipred, a well-known tool to predict sequential B-cell epitopes. Peptides with an average Bepipred score above 0.5 and a length of 9-17 amino acid residues were selected. This initial prediction step, performed separately for each species, resulted in 14,373 peptides from *A. platys* and 15,021 from *E. canis*. These peptides were then clustered on the basis of sequence similarity, irrespective of the corresponding species. The highest-scoring peptides from each cluster were then selected to build a combined prediction set for the two species. This combined set encompassed 28,805 peptides, of which 408 were shared between *A. platys* and *E. canis*. The distribution of average Bepipred scores was similar among these shared peptides and those suspected to be species-specific, with median scores around 0.56 in all cases (**Supplementary Figure S1**).

### 3.2 High-throughput identification of immunoreactive peptides

In order to experimentally validate in silico predictions of B-cell epitopes, we used a high-throughput strategy based on peptide microarray chips. Due to the limited capacity of microarrays, we prioritized peptides shared between the two species and further completed the total capacity of the chip with the top-scoring potentially species-specific peptides in the combined prediction set, including 2,244 peptides from *A. platys* and 2,348 from *E. canis*. The distribution of Bepipred scores in the set of prioritized peptides was similar to that of the entire set, with a slightly higher median of 0.57 (**Supplementary Figure S1**). A total of 5,000 peptides were printed in duplicate (10,000 spots), and the resulting microarrays were subsequently screened with three pooled canine serum samples labelled S1, S2, and S3, as well as sera from unexposed laboratory-bred dogs used as negative control. Samples S1, S2, and S3 were positive for both *A. platys* and *E. canis*, but showed varying degrees of positivity to each species, with S1 showing the highest positivity to *E. canis* and S3 the highest to *A. platys*.

**Figure 1:**
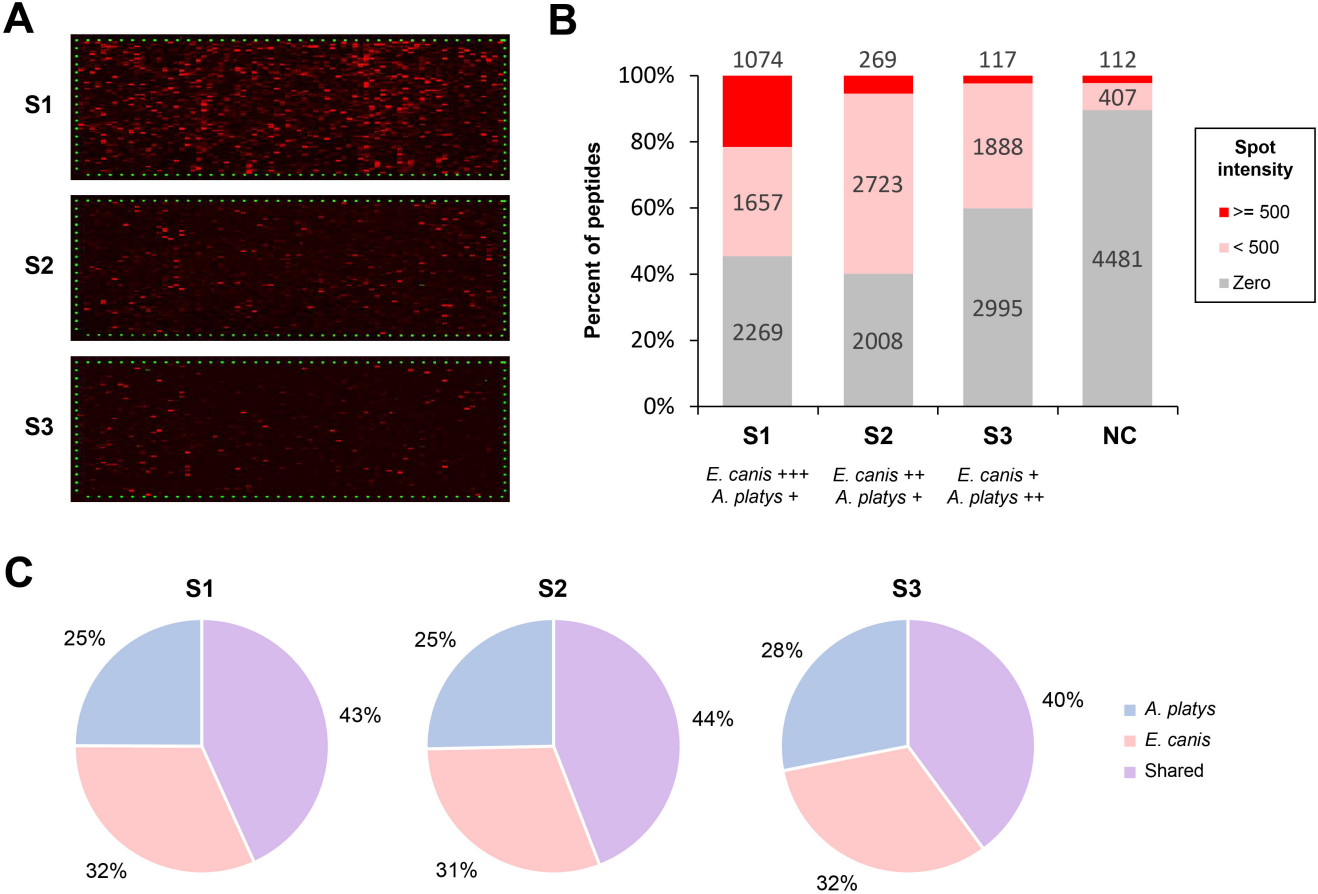
High-throughput evaluation of immunoreactivity of predicted peptides. **(A)** Screening of the prepared peptide microarray chips with three canine serum samples (S1, S2 and S3). Immunoreactive peptides appear as spots colored with different shades of red, with brighter red indicating a higher value of spot intensity. The additional HA and poliovirus control peptides framing the peptide microarrays are shown as green dots. **(B)** Bar charts showing the fraction of peptides with non-zero spot intensities for each experiment, with a threshold of 500 to indicate strong reactivity. The degree of positivity of each serum sample to each species is shown as ‘+’, ‘++’, and ‘+++’, which indicate low, moderate, or strong positivity for that species, respectively. **(C)** Fraction of potentially immunoreactive species-specific peptides and those shared between both species after screening with the three serum samples.

We observed moderate to very strong and globally complex antibody responses against multiple peptides at high signal-to-noise ratios with samples S1, S2, and S3 (**Figure 1A**). To ensure consistency in the intensity values reported for each serum sample, a spot-to-spot deviation was calculated between the two spots corresponding to each peptide in the microarrays, and the intensity was set to zero for all peptides showing a deviation above 40% (*n* = 1,182 for S1, *n* = 940 for S2, and *n* = 2,129 for S3). As expected, the overall degree of positivity of each serum sample directly influenced the global magnitude of spot intensities measured on microarrays, with a very strong response for S1, a strong response for S2, and a moderate response for S3. Globally, 40-60% of all peptides showed non-zero spot intensities for samples S1, S2 and S3, but this percentage was notably lower (~10%) for the serum sample used as negative control (**Figure 1B**). The number of peptides with relatively high intensity values varied among samples, and some of these highly reactive peptides were not predicted with the highest Bepipred scores, although they were all above the 0.5 score value recommended by the authors as a cut-off for selection (Jespersen et al., 2017).

To better describe these results, we chose a spot intensity cut-off of 500 to indicate strong reactivity, which roughly represents the lowest value of intensity observed among the top 20% ranked peptides after screening with S1. A total of 1,271 peptides showed intensity values above this cut-off for at least one serum sample, including 1,074 for S1, 269 for S2, and 117 for S3 (**Figure 1B**). Although the number of peptides strongly reacting against the negative control serum sample (*n* = 112) was numerically comparable to that for sample S3 (*n* = 117), only ten peptides were shared between the two sets. Likewise, minimal overlap was observed between samples S1 and S2 and the negative control, with only 55 peptides showing cross-reactivity between the latter and at least one of these two samples. All the peptides exhibiting cross-reactivity with the negative control serum sample, above the selected cut-off for spot intensity, were discarded from subsequent analyses (**Supplementary Table S1**).

The number of shared and species-specific immunoreactive peptides was also consistent with the degree of positivity of the serum samples for each species. For instance, results for serum sample S1 (high *E. canis* positivity) exhibited strong overall reactivity with *E. canis* peptides, whereas those for S2 (moderate *E. canis* positivity) showed comparatively lower reactivity. Although the highest values of spot intensity for these two serum samples were around 65,000, the highest ones for S3, which has weak positivity for *E. canis*, were close to 20,000. Due to their relatively higher positivity to *E. canis*, results for S1 and S2 appear to favor shared peptides or those specific to this species (**Figure 1C**). Nevertheless, several *A. platys*-specific peptides showed relatively high reactivity to all three serum samples, and their number was slightly higher for S3, which had the highest degree of positivity for this species.

After discarding peptides showing cross-reactivity with the negative control, the total number of peptides strongly reacting with at least one serum sample was reduced to 1,220 (**Supplementary Table S2**). Hereafter, this set is referred to as the immunoreactive peptides. Among them, 82 peptides were shared between the two species with nearly identical sequences.

**Figure 2:**
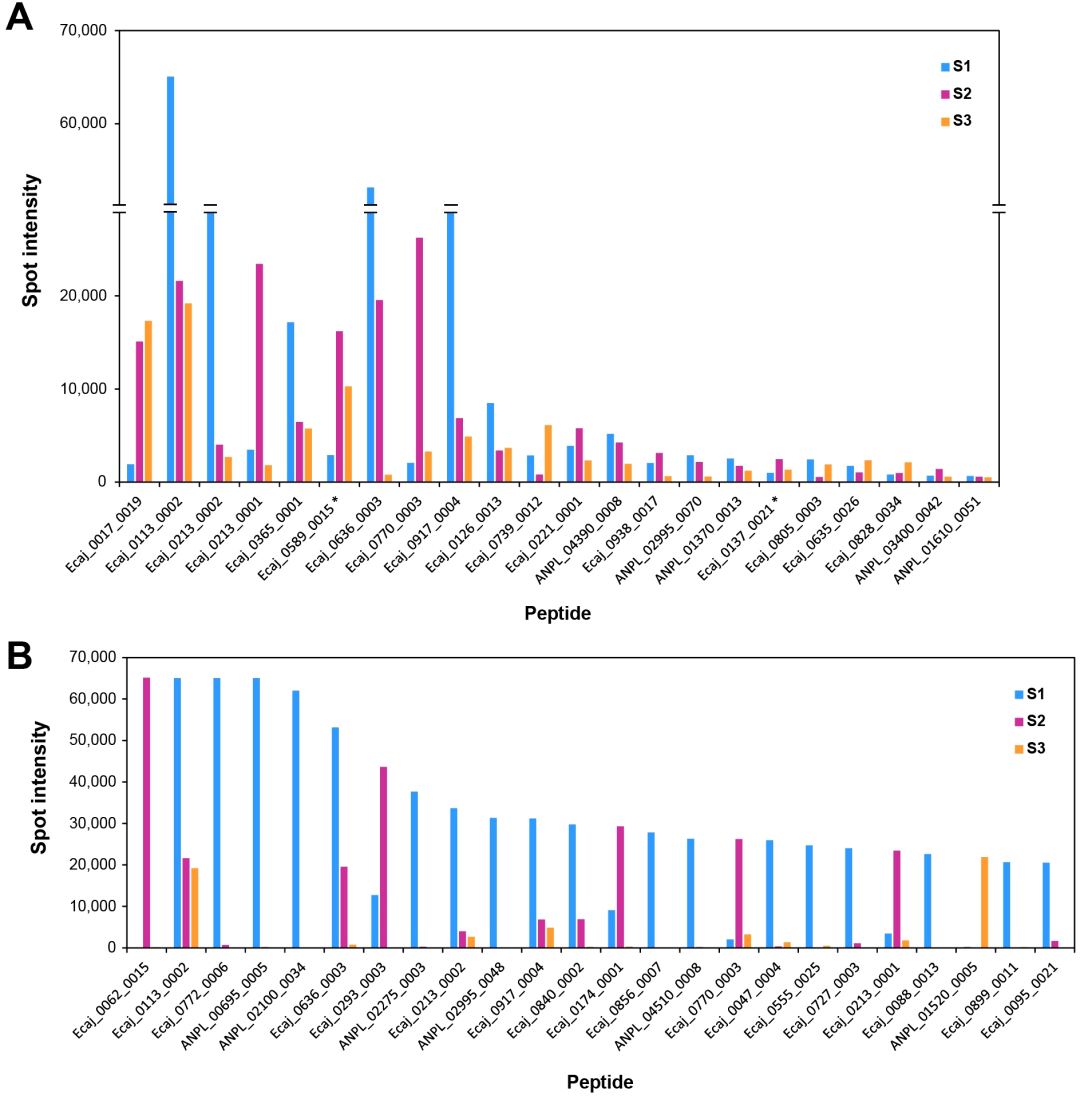
Peptides exhibiting strong reactivity in high-throughput microarray screening experiments. **(A)** Peptides strongly reacting with the three serum samples, using a threshold of spot intensity of 500 to indicate strong reactivity. **(B)** Peptides exhibiting markedly high reactivity with at least one serum sample, indicated by spot intensity values close to or above 20,000. Peptides shared by *A. platys* and *E. canis* are indicated with asterisks (*) in both panels. These peptides are labelled after the *A. platys* or *E. canis* protein in which they were first found; the corresponding peptide in the other species can be found in **Supplementary Table S2**.

As mentioned earlier, the specific set of peptides exhibiting strong reactivity differed notably for each serum sample, with minimal overlap observed among the three experiments. For instance, only 22 peptides reacted strongly with the three sera (**Figure 2A**), while 132 reacted strongly with at least two of the sera. Furthermore, a relatively small set of the peptides exhibited markedly high reactivity, indicated by values of spot intensity above or close to 20,000 (**Figure 2B**). The majority of these peptides showed these high intensity values only when reacting with S1 (*n* = 18), rather than S2 (*n* = 6) or S3 (*n* = 1). Only one peptide consistently exhibited intensity values near 20,000 for the three sera (Ecaj_0113_0002). This peptide is derived from outer membrane protein P19 (Ecaj_0113), one of the first immunodominant proteins characterized in *E. canis* (McBride et al., 2007). Similarly, several other immunoreactive peptides come from proteins already known to be highly immunogenic, such as those encoded by members of the *p44*/*msp2* superfamily (*n* = 12) and ankyrin-repeat-containing proteins (*n* = 31). Other peptides were derived from well-studied surface proteins such as type IV secretion system proteins (*n* = 22) and membrane transporters (*n* = 32). However, a notable number of immunoreactive peptides seem to come from metabolic and DNA-processing enzymes, not expected to be exposed on the cell surface. Additionally, a 25% of the immunoreactive peptides seem to be derived from proteins of unknown function.

### 3.3 Characterization of immunogenic proteins

We were also interested in characterizing the proteins contributing to the immunoreactive peptides identified through high-throughput microarray screening, which could be considered as immunogenic proteins from *A. platys* and *E. canis*. To make results comparable between both species, we first identified genes that may have derived from a common ancestor of these two species and may retain similar functions, known as orthologs. To facilitate subsequent analyses, we further clustered genes present in multiple diverging copies, such as those from the *p44*/*msp2* superfamily, which are more likely to have originated by duplication within the same genome and are therefore considered paralogs. Immunoreactive peptides, and their homologs in the case of shared peptides, were then grouped using ortholog information. Cumulative peptide counts and spot intensities were then calculated for each ortholog group to help rank the underlying proteins. This analysis resulted in 582 ortholog groups containing proteins with at least one immunoreactive peptide, of which 57 were unique to *A. platys* and 92 were unique to *E. canis*, but the vast majority (*n* = 432, 74%) were shared between both species (**Supplementary Table S3**).

Consistent with our preliminary observations about protein function, described in the previous section, the top 20 ranked proteins (**Table 1**) include major outer membrane protein P19 of *E. canis* (Ecaj_0113), two distinct ankyrin-repeat containing proteins with conserved orthologs in *A. platys* and *E. canis* (ANPL_02860/Ecaj_0387 and ANPL_02900/Ecaj_0365), and several members of the *p44*/*msp2* superfamily, also shared between the two species. The top-ranked set also included seven representative proteins of unknown function, although Ecaj_0062 appears to have at least ten additional paralogs in the *E. canis* genome.

**Table 1:** Top-ranked potentially immunogenic proteins from *A. platys* and *E. canis*.

Rank	*A. platys* Orthologs^a^	*E. canis* Orthologs^a^	Peptide count	Cumulative spot intensity^b^	Product
1	–	Ecaj_0062 (+10)	26	144,843	Hypothetical protein
2	–	Ecaj_0113	1	105,867	Major outer membrane protein P19
3	ANPL_04305 (+2)	Ecaj_0917 (+24)	10	88,901	Surface antigen MSP4
4	ANPL_03450	Ecaj_0213	6	86,481	Hypothetical protein
5	ANPL_02860	Ecaj_0387	19	88,304	Ankyrin repeat-containing protein
6	ANPL_04255 (+7)	Ecaj_0842 (+2)	7	84,785	Type IV secretion system protein, VirB2 family
7	ANPL_01085	Ecaj_0636	1	73,478	Hypothetical protein
8	ANPL_02100	–	3	70,225	Hypothetical protein
9	–	Ecaj_0174	9	68,374	Hypothetical protein
10	ANPL_01310	Ecaj_0589	6	67,679	DNA-directed RNA polymerase subunit α
11	ANPL_00695	Ecaj_0708	2	66,457	Cytochrome *c* biogenesis protein CcmA
12	–	Ecaj_0772	1	65,762	Hypothetical protein
13	ANPL_02080	Ecaj_0293	5	63,405	Membrane protein insertase YidC
14	ANPL_02900	Ecaj_0365	6	55,111	Ankyrin repeat-containing protein GP200
15	ANPL_03685	Ecaj_0170	16	43,425	DNA-directed RNA polymerase subunit β‘
16	ANPL_02275	Ecaj_0395	4	41,342	*O*-sialoglycoprotein endopeptidase
17	ANPL_02995	–	6	41,184	Hypothetical protein
18	ANPL_04150	Ecaj_0856	4	40,505	Methionine aminopeptidase
19	–	Ecaj_0017	1	34,334	GP140
20	ANPL_02515	Ecaj_0088	5	32,320	Valyl-tRNA synthetase

a Only one ortholog is shown for each group, in those groups containing additional paralogs, their number is indicated in parentheses, and the entire list of members is shown in **Supplementary Table S3**.

b The cumulative spot intensity is calculated as the sum of spot intensities for all peptides across serum samples. This value is used here only to help rank proteins. It should not be considered a property of the corresponding proteins, since intensity was measured on the peptides rather than the entire proteins.

**Figure 3:**
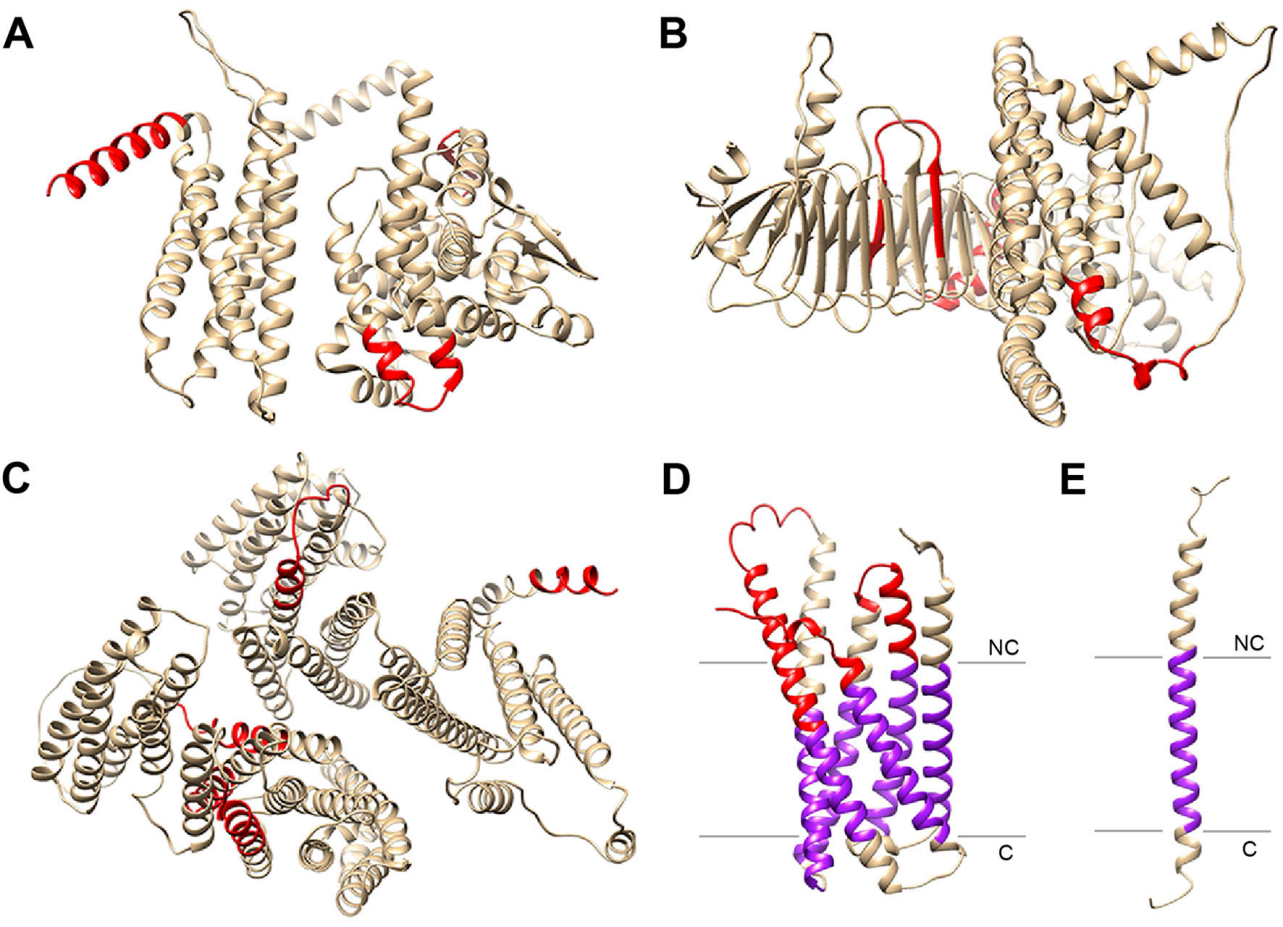
Prediction of 3D structure and membrane topology for the top-ranked immunogenic hypothetical proteins. **(A)** Ecaj_0062. **(B)** Ecaj_0174. **(C)** ANPL_02995. **(D)** Ecaj_0213 **(E)** Ecaj_0772. In all cases, only segments with relatively high local confidence and proper secondary structure elements are shown. Predicted transmembrane segments are colored in purple. Whenever possible, the location of immunoreactive peptides is shown in red. C, Cytoplasmic; NC, Non-cytoplasmic.

To better characterize these seven representative proteins of unknown function among the top-ranked immunogenic proteins, we attempted to predict their 3D structure and possible membrane topology. Predictions for ANPL_02100 and Ecaj_0636 were composed of unstructured loop regions with relatively low average pLDDT scores, and none of these proteins were predicted to contain a signal peptide or any transmembrane regions. Ecaj_0062 (residues 1-957) was predicted to be a two-domain protein (**Figure 3A**), however, structural similarity analysis using the DALI server did not reveal any reliable matches with proteins in the AlphaFold database. In turn, Ecaj_0174 (residues 1-1306) was predicted to have a four-domain topology (**Figure 3B**), in which, two of the domains share structural similarity to the core α-helices of the bacterial iron-sulfur cluster repair protein YtfE and the β-sheet arrangement of *Escherichia coli* protein YdbA, respectively. ANPL_02995 (residues 1-1471) was predicted to form six four-helix bundles (**Figure 3C**), showing high structural similarity with proteins from various organisms containing the FERM domain, involved in protein-protein and protein-membrane interactions. In the last three proteins, immunoreactive peptides were mapped to different domains. Some of these peptides were contiguously located in the sequence of the corresponding protein, suggesting predominantly linear epitopes.

Protein Ecaj_0213 (residues 1-328) was predicted to have a membrane domain formed by six α-helical transmembrane (TM) segments (**Figure 3D**). According to the AlphaFold prediction, the N-terminal region (residues 1-50), the C-terminal region (residues 303-328), and the distal ends of helices α1-α6 are oriented toward the non-cytoplasmic side. Structural similarity analysis revealed resemblance to the *E. coli* succinate-acetate/proton symporter SatP and the *Pseudomonas aeruginosa* putative transport protein AmiS. However, functional conservation between these proteins remains uncertain, as only the TM segments display limited structural similarity. Furthermore, the overall topology differs, and there is no notable similarity in the regions flanking the membrane. Most of the immunoreactive peptides from this protein seem to be located in the loops connecting TM segments and oriented to the non-cytoplasmic side of the membrane. On the other hand, Ecaj_0772 (residues 1-163) was predicted to have a single α-helical TM segment connected to a large loop, which also seems to be oriented to the non-cytoplasmic side of the membrane and contains the single, markedly immunoreactive peptide identified in this protein (**Figure 3E**).

We also compared our set of top-ranked proteins to those reported previously in a series of works aimed at identifying immunodominant proteins in *E. chaffeensis* and *E. canis* (Luo et al., 2020; Luo et al., 2021; Luo et al., 2023). Although the ranking of proteins differs, more than 70% of the *E. canis* immunodominant proteins reported by the authors were among our top-ranked proteins (**Supplementary Table S4**). We primarily identified those *E. canis* proteins reported by the authors to contain predominantly linear epitopes (Ecaj_0126, Ecaj_0213, Ecaj_0259, Ecaj_0334, Ecaj_0554, Ecaj_0636, Ecaj_0647, and Ecaj_0920). This outcome was expected since the high-throughput screening approach used in our methodology is based on sequential epitope prediction, followed by experimental evaluation using peptides rather than native proteins. However, it is worth noting that using this methodology, we were also able to identify three immunodominant proteins from *E. canis* previously reported to have mainly conformational epitopes (Ecaj_0104, Ecaj_0128, and Ecaj_0857), although these proteins were not within our top-ranked candidates. Furthermore, we also identified as immunogenic 13 *E. canis* proteins whose *E. chaffeensis* orthologs were also found to have mainly conformational epitopes (Ecaj_0018, Ecaj_0022, Ecaj_0071, Ecaj_0072, Ecaj_0172, Ecaj_0242, Ecaj_0319, Ecaj_0339, Ecaj_0349, Ecaj_0373, Ecaj_0404, and Ecaj_0513).

### 3.4 Characterization of epitopes of selected immunogenic proteins

**Figure 4:**
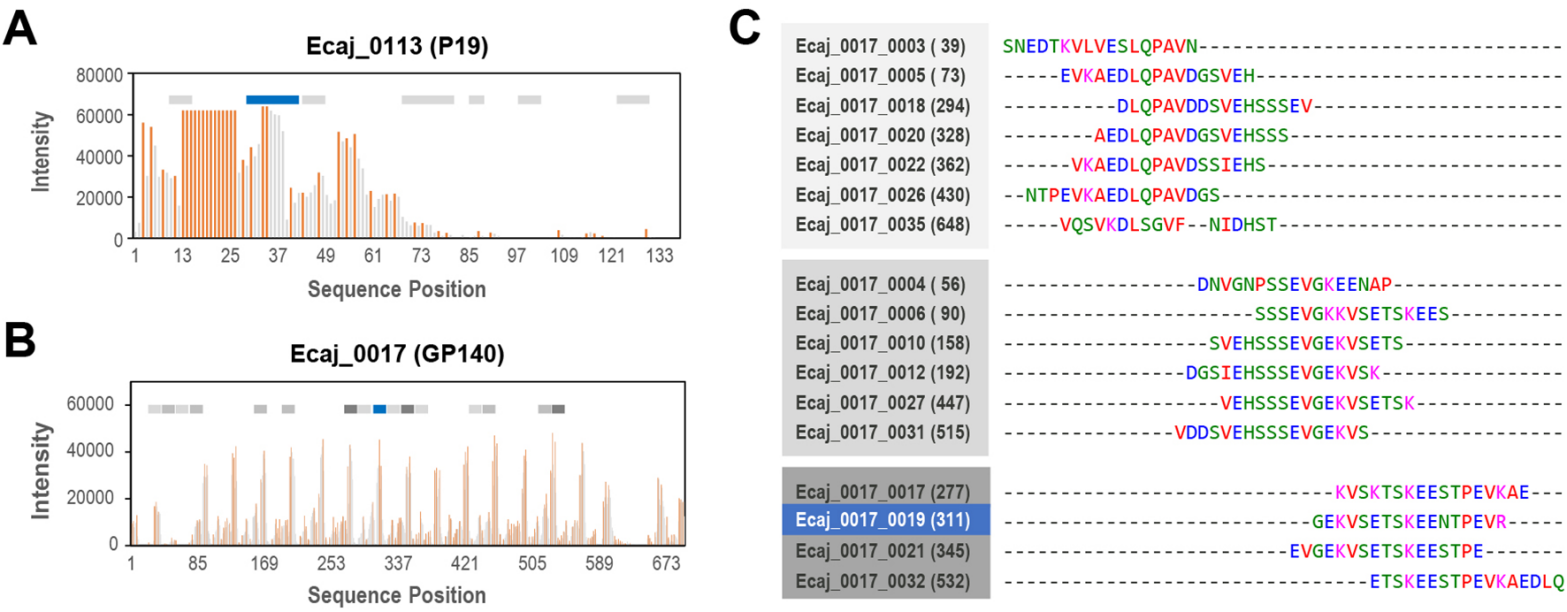
Microarray-based overlapping peptide analysis of two well-studied *E. canis* immunodominant proteins. **(A)** Spot intensity plot for Ecaj_0113 (P19) with all peptides with non-zero values plotted as gray vertical bars at their start position in sequence. Bars indicating local maxima are highlighted in orange. Horizontal bars show peptides predicted by Bepipred (gray) and those evaluated in the previous microarray screening (blue). **(B)** Similar plot for Ecaj_0017 (GP140) with predicted peptides previously clustered by sequence similarity shown in different shades of gray. **(C)** Alignment of the 17 peptides predicted for GP140. The peptides were grouped in three major clusters by sequence similarity, represented using the same shades of gray of bars from panel **(B)** and blue for the one that was experimentally evaluated. Numbers in parentheses indicate the actual position of the peptide in the sequence of the corresponding proteins.

In order to further characterize the sequential arrangement of potential epitopes in selected immunogenic proteins, we also used a high-throughput approach based on a peptide microarray chip to perform overlapping peptide analysis. To accomplish this, all the possible overlapping peptides for the eight top-ranked proteins were also synthesized on a microarray chip, totaling 4,709 peptides. Since the main goal of this step was the preliminary characterization of possible epitopes in such proteins, this microarray chip was only screened with serum sample with the strongest reactivity (S1), and the spot intensity was recorded in the same way and conditions as our previous experiments. The patterns of spot intensity variation along the sequence for *E. canis* immunodominant proteins P19 (Ecaj_0113) and GP140 (Ecaj_0017) (**Figure 4**) suggested predominantly continuous or linear epitopes, consistent with previous findings (McBride et al., 2007; Luo et al., 2009). In P19, the highest peaks of intensity were observed for overlapping peptides located within the first half of the sequence, towards the *N*-terminal end. As explained earlier, a peptide derived from this protein (Ecaj_0113_0002) was the only one reacting strongly with the three serum samples initially screened in this study. This peptide seems to be part of a larger immunoreactive region in the sequence, together with a few additional peptides with favorable Bepipred scores, which were not included in the original microarray due to its limited capacity.

Only one peptide derived from protein GP140 was also found to be immunoreactive in this study (Ecaj_0017_0019), but the overlapping peptide profile for this protein showed several intensity peaks distributed along the sequence at a regular separation (**Figure 4B**). This scenario is consistent with the architecture previously described for this protein, comprising several tandem repeats (TRs). This arrangement is composed of more or less conserved TR units repeated regularly, many of which have been shown to contain linear B-cell epitopes (Luo et al., 2009). At least 17 peptides were indeed predicted as potential B-cell epitopes by Bepipred in this protein. However, most of these peptides were clustered together on the basis of sequence similarity, as part of the clustering step we implemented to avoid redundancy and maximize the usability of microarrays. After the clustering step, these peptides were grouped into three clusters, and only a representative member of one of such clusters was included in the original microarrays. The cluster containing this peptide has three additional members that occur in close proximity to those from the other two clusters in the protein sequence, all of which seem to contribute to the TR unit (**Figure 4C**). Surprisingly, the single peptide that was prioritized for synthesis in the microarray seems to roughly correspond to the one with the highest intensity peak within its cluster in the overlapping peptide profile.

**Figure 5:**
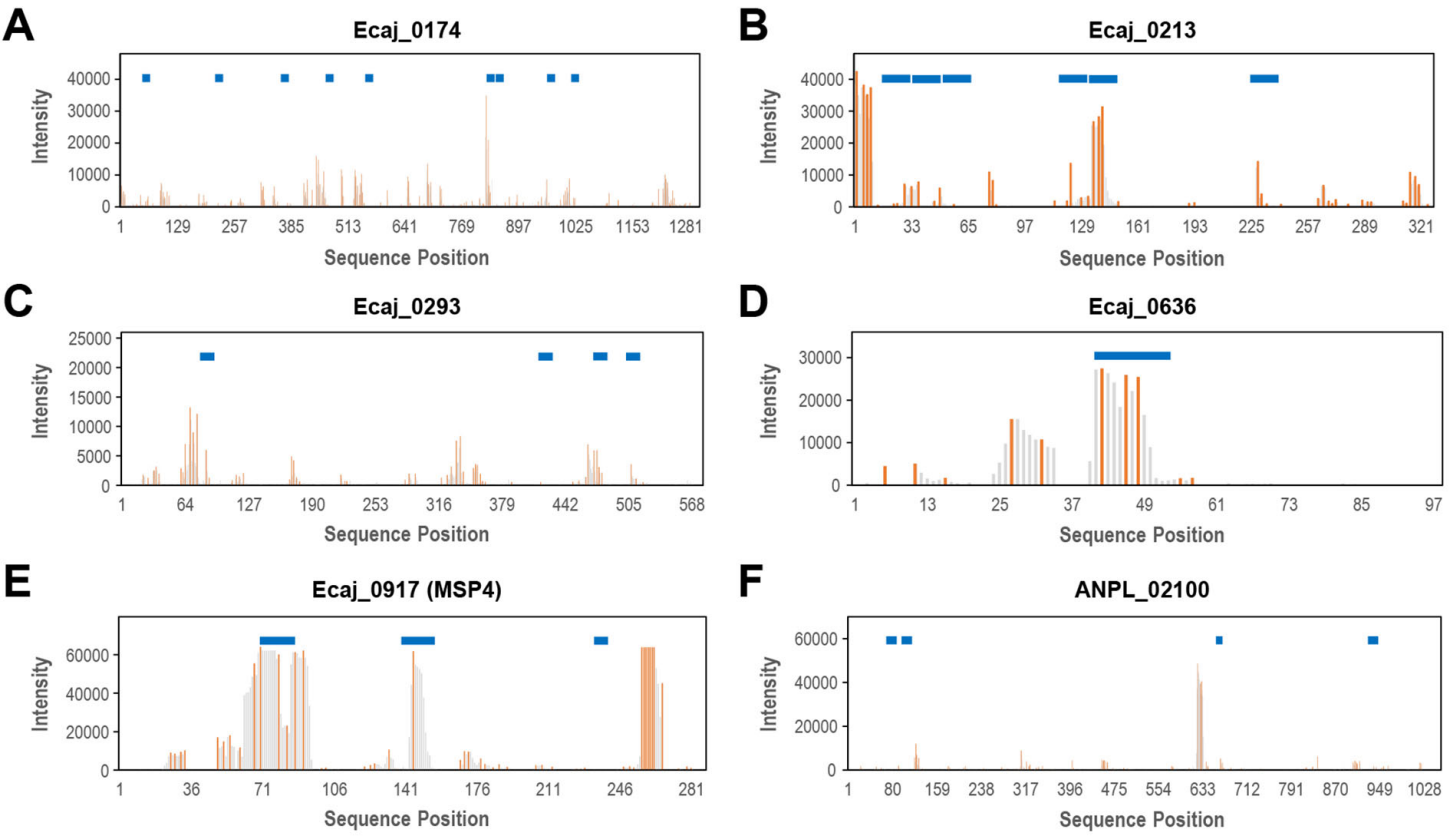
Overlapping peptide analysis for six selected immunogenic proteins. **(A)** Ecaj_0174. **(B)** Ecaj_0213. **(C)** Ecaj_0293. **(D)** Ecaj_0636. **(E)** Ecaj_0917. **(F)** Ecaj_02100. Peptides with non-zero values are plotted as gray vertical bars at their start position in the sequence. Bars indicating local maxima are highlighted in orange. Blue horizontal bars show the peptides predicted by Bepipred that were identified as immunoreactive in previous microarray screening. Plots use a different scale for both the *x* and *y* axes for each protein, to facilitate the comparison of global patterns of intensity peaks among proteins of different sequence lengths.

We observed notably different scenarios for the remaining six proteins for which overlapping peptide analysis was conducted (**Figure 5**). Although the overlapping peptide profile for Ecaj_0174 may resemble those of the TRPs discussed above, with intensity peaks and immunoreactive peptides scattered along the sequence (**Figure 5A**), most of the potential epitopes from this protein do not share noticeable sequence similarity. Likewise, the 40 peptides predicted by Bepipred in this protein could not be further clustered based on sequence similarity. A subset of 32 of these peptides were synthesized in the original microarrays, resulting in the nine immunoreactive peptides shown in **Figure 5A**. In the partial model of the 3D structure obtained for this protein (**Figure 3B**), four of these peptides seem to be located in different structural domains, whereas the other five are located in large independent loops whose structure was considered unreliable according to AlphaFold’s local confidence scores. Due to this arrangement, this protein does not seem to contain TR epitopes. Although its epitopes could still be predominantly linear, several of its derived immunoreactive peptides seem to be part of independent domains, possibly containing conformational epitopes.

**Figure 6:**
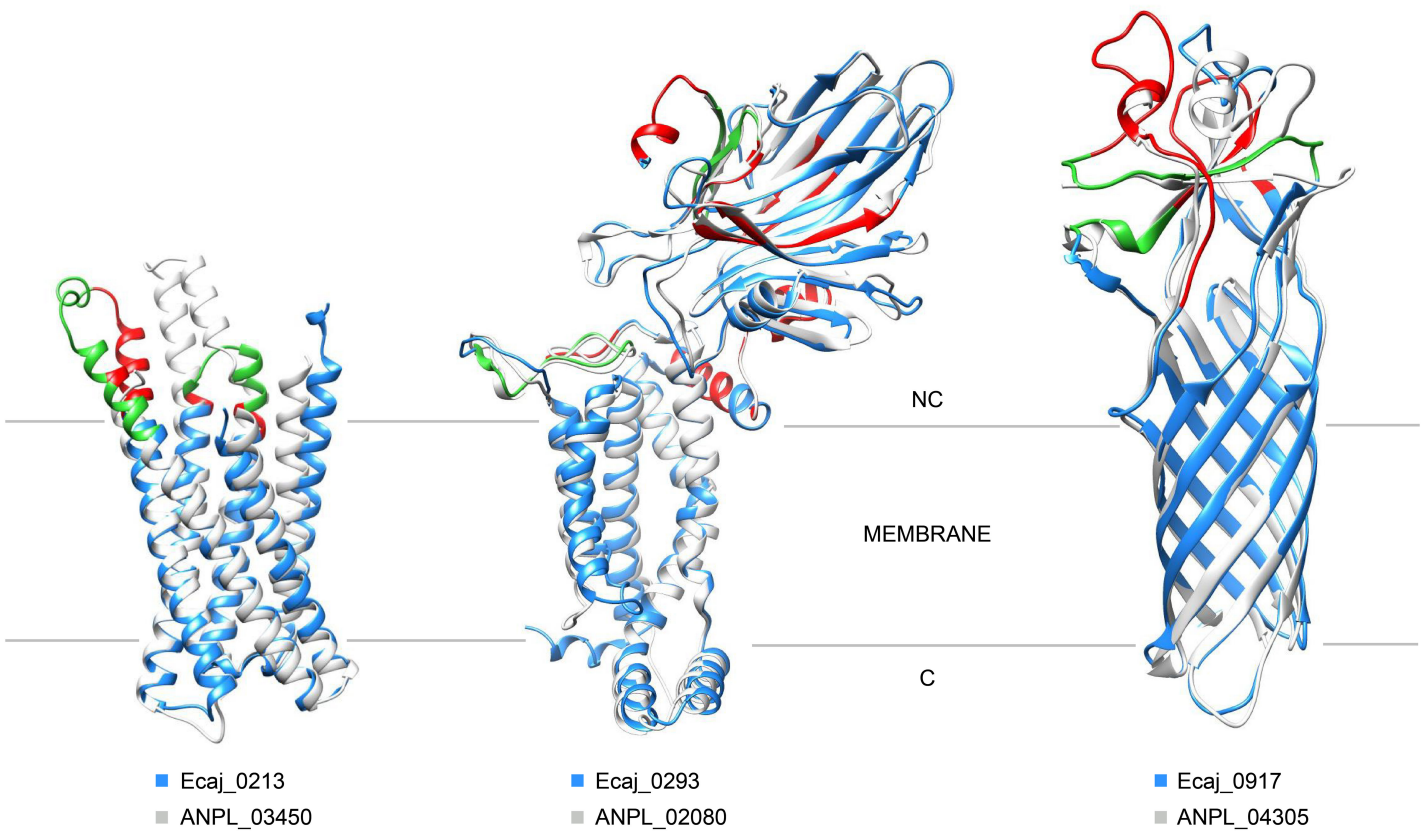
Mapping of potential epitopes from three *E. canis* putative membrane proteins on their predicted 3D models. Epitopes identified by overlapping peptide analysis are colored in red. Green indicates segments were those epitopes overlap with predicted peptides found to be immunoreactive in the previous high-throughput screening experiments. Orientation and position of each protein with respect to the membrane are approximate and were inferred by integrating TMHMM and Phobius predictions with information from conserved domains in other similar proteins, in cases where the latter information was available. Only the model segments that met AlphaFold’s criteria for high local confidence are shown.

On the other hand, the remaining five proteins for which we performed overlapping peptide analysis showed profiles characterized by intensity peaks scattered throughout the sequence, although not always with relatively high intensity values. Epitope predictions also suggested scattered peptides, many of which roughly correspond to the observed intensity peaks. We were able to model the 3D structure of three of these *E. canis* proteins and their conserved orthologs in *A. platys*, all of which appear to have a transmembrane topology (**Figure 6**). Like Ecaj_0213, whose structure was shown in **Figure 3D**, Ecaj_0293 was predicted to have an α-helical membrane domain. In contrast, Ecaj_0917 was predicted to have a β-barrel topology, typical of bacterial outer membrane porins. This protein and its ortholog in *A. platys* (ANPL_03450) belong to the *p44*/*msp2* superfamily of surface antigens, whose members in other species are indeed believed to function as outer membrane porins (Kumagai et al., 2008). In these three proteins, immunoreactive peptides seem to gather together in the non-cytoplasmic domains, despite being mostly scattered throughout the sequence. This finding does not suggest pure linear epitopes, but rather those of a conformational nature.

## 4 Discussion

*E. canis* and *A. platys* infection, transmitted by the common vector *R. sanguineous*, can cause life-threatening acute and chronic diseases in dogs. Clinical management of infected dogs typically involves antimicrobial therapy, while preventive strategies rely on tick control through the application of acaricides. However, the extensive and prolonged use of antibiotics and chemical acaricides has raised significant concerns regarding the emergence and spread of antimicrobial and antiparasitic resistance. This trend poses a critical challenge to effective disease control and underscores the urgent need for an effective vaccine. Though vaccination is an ideal practice to prevent these conditions, the development of vaccines against these pathogens is hampered by the lack of comprehensive knowledge of proteins involved in their entry, pathogenesis, and persistence in infected hosts. Identifying those proteins capable of interacting with the immune system at any stage of infection is critical for the development of vaccines and diagnostic tools. In this study, we used peptide microarray chips as a high-throughput platform to identify potential B-cell epitopes from *E. canis* and *A. platys* proteins. We also used the cumulative evidence gathered at the levels of individual peptides to characterize the underlying immunogenic proteins. The distribution of epitopes in some of these immunogenic proteins was further explored through overlapping peptide analysis, also conducted using peptide microarrays.

A subset of 1,271 peptides showed relatively high spot intensity values with at least one serum sample, of which, 51 showed cross-reactivity with the negative control serum sample and were discarded for subsequent analyses. The remaining 1,220 peptides were further considered immunoreactive, including 82 shared by both species with almost identical sequences. Relatively little overlap was observed among the set of highly reactive peptides identified after screening with the three serum samples. Similar results have been obtained in previous studies performed with immunoreactive proteins from *Ehrlichia* spp., which reported high variability in the responses of multiple human and canine sera to specific immunogenic proteins (Luo et al., 2020, Luo et al., 2021, Luo et al., 2023). However, several of the highly-reactive peptides identified in this study derived from well-known *E. canis* and *A. platys* immunodominant proteins. In *E. canis*, these include major outer membrane protein P19, ankyrin-repeat-containing proteins, and several other tandem repeat proteins (TRPs). In *A. platys*, it is worth mentioning MSP4 and other proteins from the *p44*/*msp2* gene family, considered as the major immunodominant surface proteins in all *Anaplasma* species and also present in *Ehrlichia*. Some of these proteins are routinely used in commercial diagnostic assays and have been evaluated as potential vaccine candidates targeting *E. canis* (see Alves-Ribeiro et al. (2024) for a comprehensive review), and to a lesser extent, *Anaplasma* spp. (de la Fuente et al., 2022; Moraga-Fernández et al., 2024). Most of these proteins have been reported to contain predominantly linear or sequential epitopes arranged contiguously, and in the case of the TRPs, repeated regularly along the protein sequence.

Recent studies have identified several additional immunodominant proteins from *E. canis* primarily containing conformational epitopes (Luo et al., 2020, Luo et al., 2021, Luo et al., 2023), many of which have unknown functions. In this study, we also identified immunoreactive peptides derived from such proteins in both *E. canis* and *A. platys*, despite using a methodology focused on linear epitope prediction and experimental evaluation of peptides rather than entire proteins. Unlike linear epitopes, conformational epitopes are formed by amino acid residues that are located far apart in the protein sequence, but come together after protein folding. Due to this discontinuous arrangement, conformational epitopes are determined mainly by their 3D structure rather than their sequence. These epitopes are generally identified through costly and cumbersome experiments in which immunoreactivity is measured on native proteins, but it is lost when experiments are performed with the denatured proteins. Therefore, it has been traditionally thought that conformational epitopes should be better predicted with bioinformatic tools based on 3D structures rather than primary sequence. However, Bepipred implements a machine learning algorithm trained on experimentally characterized conformational epitopes, and their authors have demonstrated that these improvements facilitates the prediction of such epitopes from protein sequences (Jespersen et al., 2017).

Prediction of conformational epitopes using protein sequences can also be justified by the fact that, although the majority of epitopes in proteins seem to be conformational, the relatively few amino acids that actually interact with antibodies are not isolated in the sequence, but indeed located within local linear stretches (Kringelum et al., 2013). Following this principle, we also found evidence of potentially conformational epitopes when mapping the results of overlapping peptide analysis to the predicted 3D structure of certain immunogenic proteins. For instance, an *E. canis* protein from the *p44*/*msp2* family (Ecaj_0917), with a conserved ortholog in *A. platys* (ANPL_04305), contains several highly reactive peptides that are scattered throughout the sequence but seem to converge in the predicted non-cytoplasmic domain of the protein, mainly composed of loops connecting β-strands of the β-barrel. A similar scenario was observed for proteins Ecaj_0213/ANPL_03459 and Ecaj_0293/ANPL_02080, which were also predicted to be membrane proteins and have scattered immunoreactive peptides mapped to putative non-cytoplasmic domains. Ecaj_0293/ANPL_02080 are homologs of the YidC protein from *Escherichia coli*, a protein that acts as a chaperone and insertase for other membrane proteins. As it was predicted for our candidates, YidC 3D structure is composed of a very small cytoplasmic domain, a membrane domain formed by five α-helical TM segments, and a periplasmic (P1) domain mainly formed by anti-parallel β-sheets (Kumazaki et al., 2014). It was in this periplasmic P1 domain, thought to act as a binding site for substrate proteins, were most of our immunoreactive peptides were located.

Certain immunodominant *E. canis* proteins containing predominantly conformational epitopes were identified as immunogenic in our study, but did not rank among our top candidates. This is likely due to the fact that our methodology identifies immunogenic proteins based on the cumulative evidence from their constituent immunoreactive peptides. Since many of these peptides seem to be part of conformational epitopes, it is possible that individual peptides may not be enough to develop the strong reactivity reported when evaluating entire proteins. In addition, evaluation of entire proteins may also imply that multiple epitopes will be exposed at once, thus guaranteeing a stronger response. Nevertheless, we believe that the identification of proteins containing mainly conformational epitopes using cost-effective, high-throughput peptide microarrays is a valuable alternative in the early stages of vaccine development. Furthermore, many of these epitopes will have value in developing improved methods for detection and differentiation between pathogens.

Effective immune protection against intracellular pathogens such as *E. canis* and *A. platys* requires a multifaceted approach, incorporating both humoral (antibody-mediated) and cellular (T-cell mediated) responses. The ideal vaccine against these pathogens should elicit a robust immune response, stimulating antibody production, cytotoxic T-cell activity to eliminate infected cells, and helper T-cell activity to enhance antigen presentation and pathogen removal. The number of epitopes identified in this study was numerous, and our future studies will streamline the most ideal proteins and will investigate these epitopes to serve as T-cell epitopes using proliferation assays. Such epitopes with the indiscriminate ability to serve as B- and T-cell epitopes can be assembled in vaccine formulations.

In summary, this study demonstrates the utility of high-throughput peptide microarrays for identifying immunoreactive B-cell epitopes from *A. platys* and *E. canis*. By mapping these epitopes to their respective proteins and exploring their distribution, we provide valuable insights into immunogenic regions, including both linear and potentially conformational epitopes. These findings lay groundwork for rational vaccine design and improved diagnostic tools. Future work will prioritize the most promising candidates for experimental validation as B- and T-cell epitopes, possibly enabling the development of multivalent vaccines that elicit robust humoral and cellular immune responses against these life-threatening canine pathogens.

## Data Availability

The original contributions presented in the study are included in the article/**Supplementary Material**. Further inquiries can be directed to the corresponding author.
