# Supplementary material for: High-throughput identification of immunoreactive peptides and corresponding proteins from Anaplasma platys and Ehrlichia canis using peptide microarray chips
Source: Front Cell Infect Microbiol. 2026 Jan 7;15:1671309. doi: 10.3389/fcimb.2025.1671309 (PMC12819751; doi:10.3389/fcimb.2025.1671309)
Supplement: Supplementary file 1 [file Image1.pdf]

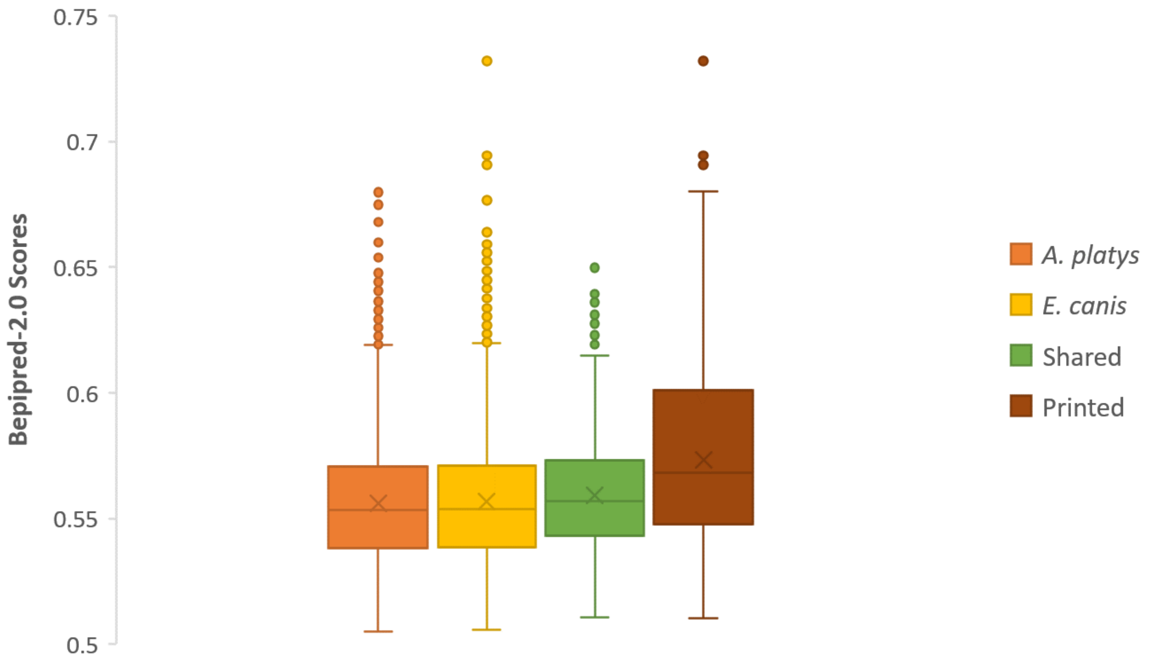

Figure S1. Distribution of Bepipred scores among predicted peptides. Distribution is represented in conventional box-and-whisker plots for peptides potentially specific to each species or shared between them, as well as those selected for printing on the microarray chips. Peptides with markedly high scores are shown as dots above the upper whisker.
