# Supplementary material for: High-throughput identification of immunoreactive peptides and corresponding proteins from Anaplasma platys and Ehrlichia canis using peptide microarray chips
Source: Front Cell Infect Microbiol. 2026 Jan 7;15:1671309. doi: 10.3389/fcimb.2025.1671309 (PMC12819751; doi:10.3389/fcimb.2025.1671309)
Supplement: Supplementary file 5 [file Table4.pdf]

Supplementary Table 4. Comparison of our results with previously characterized immunodominant proteins from *E. chaffeensis* and *E. canis*

Rank[a]	*E. canis* Orthologs[b]	*E. chaffensis* Orthologs[b]	*A. platys* Orthologs	Epitopes[c]	Product
1	Ecaj_0071<br>Ecaj_0072	**Ech_0121**	**-**	C	Hypothetical protein
5	**Ecaj_0213**	**-**	ANPL_03450	L	Hypothetical protein
7	**Ecaj_0636**	Ech_0377	ANPL_01085	L	Hypothetical protein
42	**Ecaj_0348**	**-**	-	C	Hypothetical protein
50	**Ecaj_0554**	Ech_0471	ANPL_01480	L	Heat shock protein Hsp70
53	**Ecaj_0104**	Ech_0159	-	C	Hypothetical protein
61	**Ecaj_0647**	Ech_0365	ANPL_01055	L	Chaperonin Cpn60/TCP-1
66	**Ecaj_0126**	Ech_0187	-	L	Hypothetical protein
79	Ecaj_0319	**Ech_0755**	ANPL_02200	C	Sensor histidine kinase
86	Ecaj_0018	**Ech_0040**	ANPL_00190	C	Type IV secretion system protein VirD4
130	**Ecaj_0259**	Ech_0825	-	L	Hypothetical protein
152	**Ecaj_0920**	Ech_1148	-	L	Hypothetical protein
162	Ecaj_0373	**Ech_0670**	**-**	C	Hypothetical protein
181	**Ecaj_0857**	Ech_1065	ANPL_04145	C	2-oxoglutarate dehydrogenase E2 component
225	**Ecaj_0151**	Ech_0976	ANPL_03790	L	Electron transport protein SCO1/SenC
229	**Ecaj_0334**	Ech_0731	ANPL_02470	L	PpiC-type peptidyl-prolyl cis-trans isomerase
244	Ecaj_0242	**Ech_0846**	ANPL_03175	C	Hypothetical protein
273	Ecaj_0022	**Ech_0044**	ANPL_00210	C	Type IV secretion system protein VirB8
286	Ecaj_0172	**Ech_0947**	**-**	C	Hypothetical protein
294	Ecaj_0513	**Ech_0518**	**-**	C	Hypothetical protein
367	Ecaj_0404	**Ech_0635**		C	Hypothetical protein
426	Ecaj_0339	**Ech_0725**	ANPL_02375	C	Hypothetical protein
484	Ecaj_0349	**Ech_0706**	ANPL_01400	C	Hypothetical protein
497	**Ecaj_0128**	Ech_0189	ANPL_00425	C	Extracellular solute-binding protein, family 1

[a] Rank among the 582 ortholog groups suspected to contain immunogenic proteins in this study (see Table S3).
[b] As reported by the works by Luo et al. (see main text). The protein for which immunogenicity was experimentally measured is highlighted in bold.
[c] Predominant nature of epitopes, as reported by the works of Luo et al., C: conformational, L: Linear.
